# The effect of teaching style and academic motivation on student evaluation of teaching: Insights from social cognition

**DOI:** 10.3389/fpsyg.2022.1107375

**Published:** 2023-01-19

**Authors:** C. Keerthigha, Smita Singh

**Affiliations:** School of Social and Health Sciences, James Cook University, Singapore, Singapore

**Keywords:** warmth, competence, task-oriented, relationship-oriented, student evaluation of teaching, student academic motivation, social cognition, mediation

## Abstract

Student evaluation of teaching (SET) is ubiquitous in higher education as a metric for assessing teachers, gaining student feedback, and informing faculty personnel decisions. It is thus imperative to examine the dimensions along which a teacher is judged. This study tested the application of the universal dimensions of social judgment (i.e., warmth and competence) in SET. A total of 108 psychology undergraduates (*M*_age_ = 23.63, *SD*_age_ = 3.14) in Singapore rated a fictitious teacher (i.e., either relationship-oriented or task-oriented) based on their interactions over a programmed online chat. Participants responded to the social judgment measures of warmth and competence and rated their academic motivation. Results indicated a higher SET rating for a relationship-oriented than a task-oriented teacher. Further, student academic motivation mediated the link between teaching style and judgment of competence. The findings extend the supremacy of warmth in the context of SET, thus supporting the application of social cognition literature to educational research. In addition, the findings suggest that fostering a match in task goals between a teacher and student improves ratings of teacher competence.

## 1. Introduction

To improve quality teaching, regular and objective examination of teachers is imperative (Spooren et al., 2013). Since the 1920s, universities have relied on students to assess teachers. Students are considered relevant stakeholders in gathering insights into teaching quality. SET is primarily drawn on the perception of teaching style, and the experience one has with the teacher (Coldren and Hively, 2009). SET is a tool for measuring teaching performance either in whole or part (Spooren et al., 2013). However, the basis of these perceptions has yet to be thoroughly investigated (Zhao et al., 2022). There is thus merit in extending the decades of research in social cognition to the domain of SET. Research has established *warmth* and *competence* as the two universal dimensions of social perception (Fiske et al., 2007). In the present study, we address the possibility of applying the tenets of social judgment to the parameters of SET.

### 1.1. Teaching style

Teaching style refers to a pervasive quality of teaching behavior that persists even though the taught content changes (Ghanizadeh and Jahedizadeh, 2016). Teaching style has been documented to affect student learning experience and student impressions of the teacher (Coldren and Hively, 2009), potentially factoring into SET. Like leaders, teachers influence students’ attitudes and behavior (Yukl, 1989). Teachers monitor, motivate, manage, and engage students. Their expertise grants them respect and authority in the classroom to control rewards and punishment for students. These functions draw parallels between a teacher and a leader. A teacher’s impact on the education system is synonymous with a leader’s role in organizational success. Hence, a teacher’s position mirrors a leader’s hierarchical power structures in a high power distance organization (Ryan et al., 2017).

The task-relationship model in organizational psychology distinguishes leader behavior as either work oriented and achievement focused (task) or person oriented and relationship focused (relationship; Northouse, 2018). Therefore, task-oriented leaders prioritize goal attainment by efficiently allocating resources and delegating responsibilities to their followers. Relationship-oriented leaders help followers feel comfortable with the self, others, and the situation (Cohen et al., 2004). In education, teachers who embody a task-oriented style demand high academic performance by providing rigorous instructions and challenges to students (Sandilos et al., 2017). On the other hand, teachers who embody a relationship-oriented style render warmth to students through unconditional positive regard, attentiveness, care, and respect. Adopting an appropriate teaching style is integral to good teaching practices and evaluation. Thus, the present study investigated the applicability of two leadership styles in teaching and how they may affect SET.

### 1.2. Student evaluation of teaching

Adhering to Spooren et al. (2013) recommendations for designing SET, there is merit in applying the well-established concept of social judgment to SET. Research in social cognition suggests that warmth and competence are universal dimensions based on how we perceive and relate to others. According to the stereotype content model (SCM), the universality of the dimensions results from one’s need to survive and thrive in the social world (Fiske et al., 2007). The judgment of warmth anticipates others’ intentions toward us and is accompanied by questions of their trustworthiness, sincerity, kindness, and friendliness (Aaker et al., 2010). Next, in temporal sequence, the judgment of competence anticipates others’ capability to enact those intentions through their demonstrations of respect, self-efficacy, skills, confidence, and intelligence. The SCM’s generality across place, levels, and time (Fiske, 2018) further supports the application of the social judgment dimensions to SET.

Purportedly, warmth corresponds with traits related to relationship-orientation, while competence coincides with task-orientation (Brambilla et al., 2010). However, this begs the question of whether a task-oriented teaching style is perceived as higher on competence than a relationship-oriented teaching style; and whether a relationship-oriented teaching style is perceived as higher on warmth than a task-oriented teaching style. Thus, the main research question we investigate is whether the evaluation of task and relationship-oriented teaching styles differs on the dimensions of warmth and competence.

*Hypothesis 1*: There would be a significant main effect of teaching style on the SET dimensions of warmth and competence.

*Hypothesis 1a*: A task-oriented teacher would be rated higher on competence than a relationship-oriented teacher.

*Hypothesis 1b*: A relationship-oriented teacher would be rated higher on warmth than a task-oriented teacher.

### 1.3. Student academic motivation

Student academic motivation is the vigor to engage, learn, and work effectively to achieve potential (Martin, 2010). Komarraju (2013) revealed that students who lacked academic motivation valued the ‘caring’ trait in a teacher, while motivated students strongly endorsed the importance of a teacher being more professional than caring. Hence, the finding implies that students with high academic motivation prefer a task-oriented teacher, while those with low academic motivation prefer a relationship-oriented teacher. This speculation calls into question the role of student academic motivation when evaluating the two teaching styles in the present study. According to Dignath-van Ewijk (2016), a match in task goals between two individuals forms the basis for assessing competence. Given that student academic motivation and teacher competence are grounded in the same need for task achievement (Guay et al., 2010), this study hypothesizes that academic motivation would control how students perceive the respective teaching styles on the SET dimension of competence.

*Hypothesis 2*: Student academic motivation will mediate the effect of teaching style on the SET dimension of competence.

## 2. Present study

In applying the social judgment dimensions to SET, participants of the present study will evaluate the task-oriented and relationship-oriented teaching styles based on the dimensions of warmth and competence. Additionally, the present study will test for the mediating effect of student academic motivation on the relationship between teaching style and the SET dimension of competence.

## 3. Methodology

### 3.1. Participants and design

One hundred and eight psychology undergraduates in Singapore (*M*_age_ = 23.63, *SD*_age_ = 3.14) participated in the exchange of course credits. The participants were randomly assigned to one of two between-subjects experimental conditions (teaching style: task vs. relationship).

### 3.2. Measures

#### 3.2.1. Teacher judgment (DV)

Participants rated the teacher using the 12-item teacher judgment scale (Poorani and Singh, 2015; *α* = 0.94). The scale was patterned after the established social judgment dimensions (Fiske et al., 2007) of *warmth* (e.g., ‘I think this lecturer would be approachable’, ‘this lecturer would be friendly toward individual students’) and *competence* (e.g., ‘this lecturer is probably an intelligent individual’, ‘this lecturer would probably achieve all of their goals’). Participants responded on a 7-point Likert scale, ranging from 1 (*strongly disagree*) to 7 (*strongly agree*).

#### 3.2.2. Student academic motivation (MV)

The 12-item academic motivation sub-scale (Martin, 2010; *α* = 0.83) of the Motivation and Engagement Scale–University/College was used to assess participants’ academic motivation (e.g., If an assignment is difficult, I keep working at it trying to figure it out). Participants responded on a 7-point Likert scale, ranging from 1 (*strongly disagree*) to 7 (*strongly agree*).

#### 3.2.3. Manipulation check

To check the success of the experimental manipulation of teaching style, participants rated the teacher on the Least Preferred Co-worker (LPC) scale (Fiedler, 1964). Participants described the teacher on a series of 18, 8-point bipolar semantic differential scales (e.g., rejecting—accepting). The favorable pole of each scale is scored as “8,” and the unfavorable pole as “1.” Scores for all scales are summed, with low-LPC scores (i.e., 18—64) indicating task-orientation; high-LPC scores (i.e., 73—144) indicating relationship-orientation; and mid-ranged LPC scores (i.e., 65—72) indicating a hybrid. The LPC scores were matched against the participant’s assigned experimental condition.

### 3.3. Teaching style manipulation (IV) and procedure

**Figure 1:**
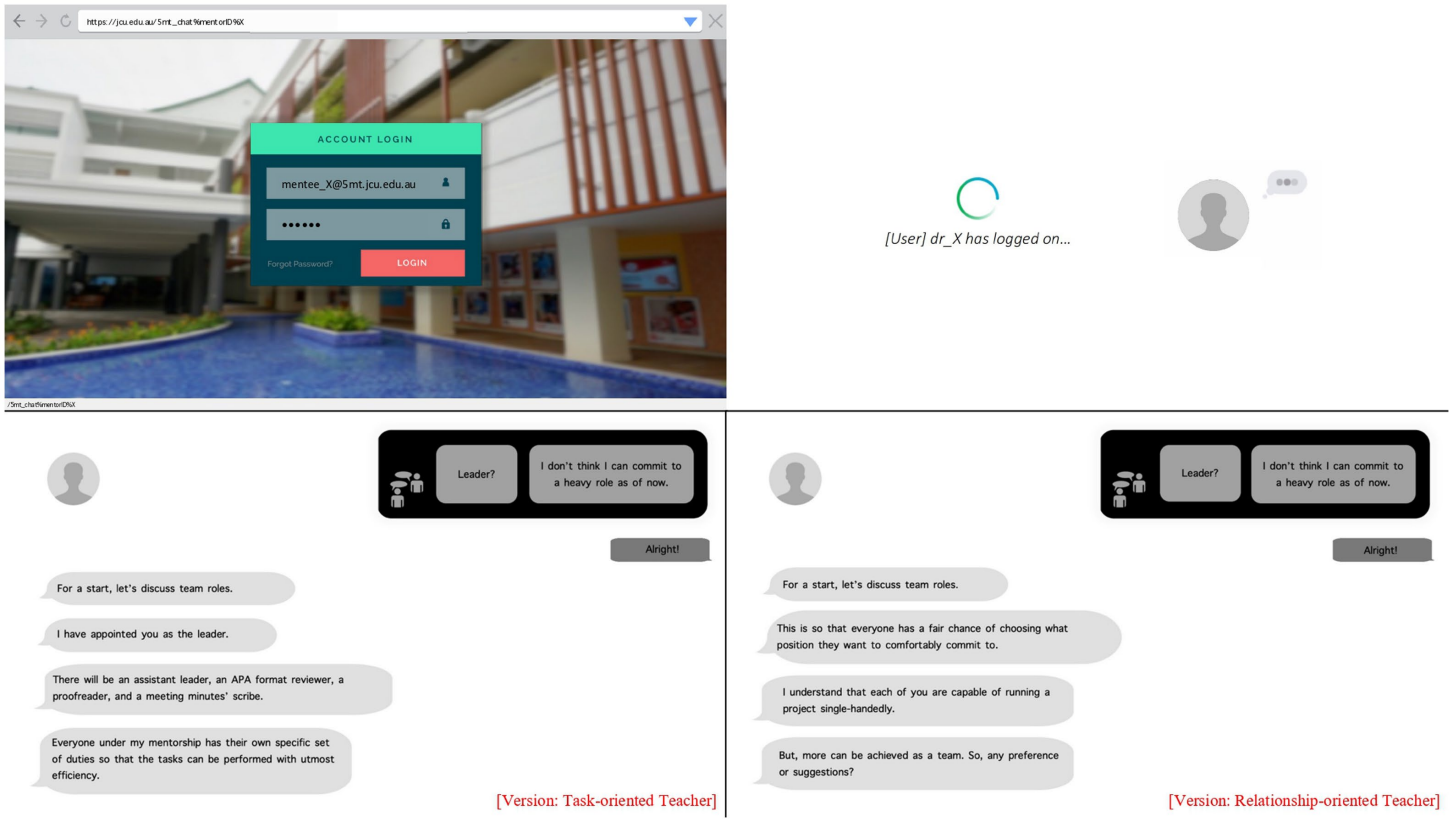
Images of the user session (i.e., the login screen of the online chat, loading, typing animations) and sample block of interaction between the (task-oriented vs. relationship-oriented) teacher and participant in the online chat.

The study was approved by the Human Research Ethics Committee at James Cook University (Ref.H7563). Upon arrival at the lab, participants were assigned to one of two experimental conditions (i.e., task-oriented vs. relationship-oriented teacher) *via* permuted block randomization. Participants read a circular introducing a fictitious teacher who would mentor their research project. The circular contained information about the teacher’s research interest, field of expertise, and years of experience. The teaching style was manipulated using an online chat programmed to facilitate interaction between the teacher and participants. The chat was presented on Microsoft PowerPoint’s kiosk mode with images of the user session and loading and typing animations (see Figure 1).

The teacher’s questions and instructions to participants were patterned after Northouse’s (2018) Style Questionnaire (*α* = 0.93). Two versions were developed to correspond to each teaching style. The relationship-oriented teacher was programmed to show flexibility in making decisions by allowing participants to choose the role they would like to be assigned for the research project. In contrast, the task-oriented teacher was programmed to pre-assign roles to the participants. Participants engaged in the online chat according to their assigned experimental condition. There was a total of six blocks of interaction. Participants took ~6 min to interact with their teacher. Participants chose one of two response choices for each block. The response options were kept the same throughout both conditions. A sample block of interaction from both versions are shown in Figure 1. At the end of the chat, participants were invited to complete all the study’s questionnaires. The study took ~20 min to complete. Participants were debriefed after they finished responding. Data analyses were performed using IBM’s SPSS version 27.

## 4. Results

### 4.1. Manipulation check

To test if the teaching style manipulation produced intended effect, an independent *t*-test was performed on the LPC scores. It revealed a significant difference between the scores in two identifiable levels of the teaching style condition (i.e., relationship-and task-orientation), *t*(106) = 20.86, *p* < 0.001, Cohen’s *d* = 4.01. Participants rated the relationship-oriented teacher (*M* = 108.31, SD = 22.04) higher than the task-oriented teacher (*M* = 33.74, *SD* = 14.31) on the LPC scale. The result was consistent with the LPC score interpretation wherein higher scores on the LPC scale indicate relationship orientation while lower scores indicate task orientation. This verified the effectiveness of the teaching style manipulation.

### 4.2. Construct distinction and reliability

To test for construct distinction among the measures of competence, warmth, and student academic motivation, a principal components analysis was conducted on all 24 items using direct oblimin rotation. Factor patterns demonstrated clear loadings on the three factors and explained 80.06% of the total variance. Table 1 lists the factor patterns in the responses.

**Table 1:** Factor patters in the responses to the competence, warmth, and student academic motivation measures.

Responses to the items	Factor 1	Factor 2	Factor 3
Factor 1: Competence (C)			
C1: This lecturer is probably a gifted individual.	−0.86		
C2: This lecturer is probably a talented individual.	−0.89		
C3: This lecturer is probably an intelligent individual.	−0.86		
C4: This lecturer would probably be successful in life.	−0.87		
C5: This lecturer would probably achieve all of her goals.	−0.81		
C6: This lecturer is probably a competent individual.	−0.76		
Factor 2: Warmth (W)			
W1: I think this lecturer would be approachable.		0.91	
W2: This lecturer seems to make students feel welcome in seeking help in or outside of class.		0.85	
W3: This lecturer seems to have a genuine interest in individual student.		0.89	
W4: This lecturer would be friendly toward individual students.		0.9	
W5: This lecturer would probably respect students as individuals.		0.82	
W6: I would enjoy discussing controversial topics with this lecturer.		0.8	
Factor 3: Student Academic Motivation (SAM)			
SAM1			0.88
SAM2			0.9
SAM3			0.9
SAM4			0.92
SAM5			0.94
SAM6			0.97
SAM7			0.95
SAM8			0.93
SAM9			0.9
SAM10			0.9
SAM11			0.96
SAM12			0.94
Variance explained (%)	47.67	21.25	11.14

In addition, the three distinct constructs showed excellent levels of internal consistency. The intercorrelations, reliability coefficients, and descriptives are presented in Table 2.

**Table 2:** Intercorrelations, reliability coefficients, and descriptive of the competence, warmth, and student academic motivation measures.

	1	2	3
1. Competence	–	–	–
2. Warmth	0.23**	–	–
3. Student Academic Motivation	0.42**	−0.05	–
Number of items	6	6	12
Cronbach’s Alpha (*α*)	0.93	0.93	0.98
*M*	4.8	5.27	5.57
SD	1.35	1.36	1.63
Actual range	1–7	1.33–7	1–7
Potential range	1–7	1–7	1–7

**p < 0.01.

### 4.3. Hypotheses testing

#### 4.3.1. Hypotheses 1, 1a, 1b

A multivariate analysis of variance (MANOVA) was used to examine the effect of teaching style on warmth and competence (*N* = 108). All underlying assumptions were supported. Findings revealed a significant main effect of teaching style on the combined DVs of warmth and competence, *F*(2, 105) = 13.64, *p* < 0.001; Wilk’s Λ = 0.79, partial *η*^2^ = 0.21, indicating that Hypothesis 1 was supported. The individual DVs were analyzed at the Bonferroni-adjusted alpha level of 0.025. There was no significant effect of teaching style on competence (*p* = 0.248), indicating that Hypothesis 1a was not supported. However, the effect of teaching style on warmth was statistically significant, *F*(1, 106) = 20.52, *p* < 0.001, *η*^2^ = 0.16, indicating that Hypothesis 1b was supported. Participants rated the relationship-oriented teacher significantly higher on warmth (*M* = 5.82, *SD* = 1.11) than the task-oriented teacher (*M* = 4.73, *SD* = 1.37).

#### 4.3.2. Hypothesis 2

Mediation analysis was performed using SPSS Process Model 4 (Hayes, 2018). Model 4 estimated (1) the indirect effect (IE) of teaching style on competence *via* academic motivation, (2) the bias-corrected 95% confidence interval (CI) around that IE from 5,000 bootstrap resamples, and (3) the mediation effect size (ES). We accept the IE as greater than zero if its bias-corrected 95% CI excluded zero.

**Figure 2:**
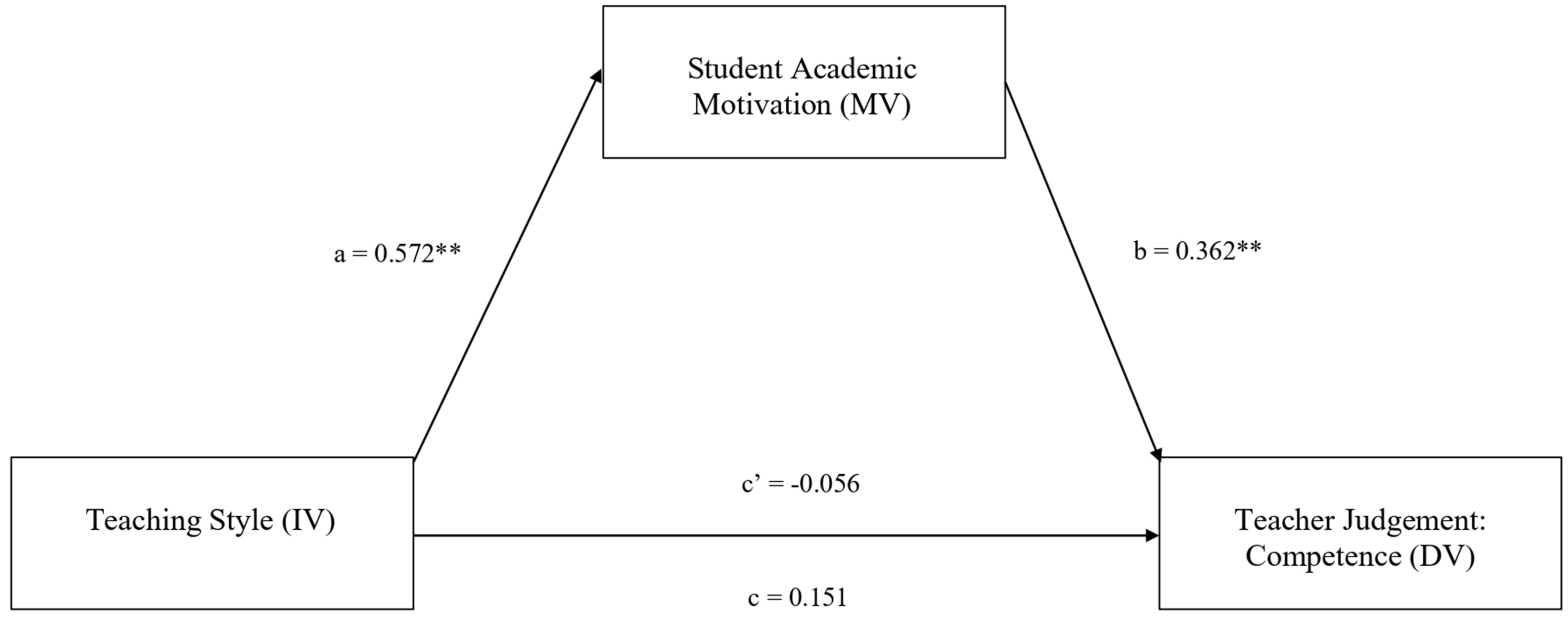
The mediating effect of student academic motivation on the relationship between teaching style and the SET dimension of competence. **p < 0.001.

The IE of teaching style on competence *via* academic motivation was significant, IE = 0.21, bias-corrected 95% CI [0.068, 0.371]. However, the direct effect of teaching style on competence was nonsignificant, *b* = −0.056, *t* = −0.437, *p* = 0.66. While a mediation in the absence of a total effect (*b* = 0.151, *t* = 1.162, *p* = 0.25) may seem contradictory, evidence has suggested that the lack of a total effect does not preclude the possibility of observing an IE (Rucker et al., 2011; Kenny and Judd, 2014). This anomaly may be attributed to the inadequate sample size of the present study. Post-hoc power analysis revealed an obtained power of 0.73 (alpha = 0.05; Faul et al., 2009). Thus, it can be argued that student academic motivation mediated the relationship between teaching style and competence, indicating that Hypothesis 2 was supported. Results are presented in Figure 2.

## 5. Discussion

The present study aimed to investigate the effect of teaching style on SET. Results indicated a higher SET rating for a relationship-oriented than a task-oriented teacher. Further, student academic motivation mediated the link between teaching style and judgment of competence.

### 5.1. Findings and implications

Hypothesis 1, which predicted a significant main effect of teaching style on the dimensions of SET, was supported. A significant multivariate effect meant that the two teaching styles were discriminated against on the linear combination of warmth and competence. This finding supported the two fundamental and distinct categories of leadership style (Northouse, 2018). The task-relationship model continued to differentiate beyond leadership literature. We can say that the online chat patterned after Northouse (2018) effectively discriminates between the two teaching styles, and teachers are perceived as leaders based on their behavior or style.

Hypothesis 1a, which predicted that a task-oriented teacher would be rated higher on competence than a relationship-oriented teacher, was rejected, implying that students perceived both teaching styles as relatively equal on the dimension of competence. Cuddy et al. (2008) argue that task-orientation is not fully representative of competence. Task-orientation focuses more on taking action, whereas competence entails possessing skills, talents, and capabilities. In the online chat, the task-oriented version of the teacher was programmed to mainly emphasize goal setting and delegation of workload (i.e., taking actions). According to Dignath-van Ewijk (2016), an individual’s competence only matters when they have personal relevance to the perceiver. In our study, a lack of information about the teacher’s distinguishable competence traits and the low importance of the task may have diminished the sense of personal relevance for the participants.

Hypothesis 1b, which predicted a relationship-oriented teacher would be rated higher on warmth than a task-oriented teacher, was supported. This was consistent with Cohen et al. (2004)‘s description of relationship orientation comprising warmth traits. Overall, findings show that the judgment of competence is constant while warmth varies across both teaching styles. This supports the supremacy of warmth over competence in that people are cognitively more sensitive to information regarding others’ warmth than competence cues (Cuddy et al., 2008). An important implication is that enacting warmth cues play a pivotal role in managing student impressions of the teacher and that warmth is specific to a relationship-oriented teaching style.

Hypothesis 2, which predicted student academic motivation would mediate the effect of teaching style on the SET dimension of competence, was supported. The mediation analysis revealed that relative to a relationship-oriented teacher, a task-oriented teacher was rated on average 0.21 (*ab*) units higher on competence due to student academic motivation. This was in line with Dignath-van Ewijk (2016) research, where a match in task goals forms the basis for student appraisal of teacher competence. This proposes that practicing a teaching style appropriate for the student’s academic motivation is pivotal for high SET scores on competence.

### 5.2. Contributions

The present study has extended the application of organizational and social cognition principles to research in education. It has not only tested but established the universality of social judgment dimensions in setting the parameters of SET. Further, findings supported the universality of the stereotype content model and have established high reliabilities for the two-factor model of warmth and competence. In addition, our study champions the adoption of the two-factor leadership models in the teaching domain. This encourages further theoretical and empirical explorations in generalizing ideas and theories developed within organizational psychology to the context of teaching.

### 5.3. Limitations and future directions

The present study was constrained to a smaller sample size with post-hoc power analysis (0.73; alpha = 0.5) falling below the recommended power of 0.8 (Faul et al., 2009), thus warranting a bigger sample size. Further, future studies could expand on the present findings by including the gender of the teacher and student as variables of interest. Understanding potential gender biases may contribute to the existing literature as extraneous factors biasing SET.

## 6. Conclusion

The present study extends the application of organizational and social psychology principles to research in the educational setting. By adopting universal dimensions of social judgment to the parameters of SET, the study has reinstated the supremacy of warmth in the SET context. Furthermore, fostering a match in task goals between a teacher and student improves ratings of teacher competence.

## Data availability statement

The raw data supporting the conclusions of this article will be made available by the authors, without undue reservation.

## Ethics statement

The studies involving human participants were reviewed and approved by the Human Research Ethics Committee, James Cook University, Australia (Ref. H7563). The patients/participants provided their written informed consent to participate in this study.

## Author contributions

CK contributed to the conception and design of the study, data collection, data analysis, and wrote the first draft of the manuscript. SS contributed to the manuscript preparation, provided advice on the design of the study and statistical methods. All authors contributed to the manuscript revision, read, and approved the submitted version.

## Funding

This research publication was funded by the Internal Research Fund, James Cook University, Singapore.

## Conflict of interest

The authors declare that the research was conducted in the absence of any commercial or financial relationships that could be construed as a potential conflict of interest.

## Publisher’s note

All claims expressed in this article are solely those of the authors and do not necessarily represent those of their affiliated organizations, or those of the publisher, the editors and the reviewers. Any product that may be evaluated in this article, or claim that may be made by its manufacturer, is not guaranteed or endorsed by the publisher.
